# What is a related work? A typology of relationships in research literature

**DOI:** 10.1007/s11229-022-03976-5

**Published:** 2023-01-09

**Authors:** Shayan Doroudi

**Affiliations:** School of Education, University of California, Irvine, 401 E. Peltason Drive, Suite 3200, Irvine, CA 92617 USA

**Keywords:** Literature search, Literature-based discovery, Information retrieval, Relevance, Analogies, Abstraction, Structure-mapping theory

## Abstract

An important part of research is situating one’s work in a body of existing literature, thereby connecting to existing ideas. Despite this, the various kinds of relationships that might exist among academic literature do not appear to have been formally studied. Here I present a graphical representation of academic work in terms of entities and relations, drawing on structure-mapping theory (used in the study of analogies). I then use this representation to present a typology of operations that could relate two pieces of academic work. I illustrate the various types of relationships with examples from medicine, physics, psychology, history and philosophy of science, machine learning, education, and neuroscience. The resulting typology not only gives insights into the relationships that might exist between static publications, but also the rich process whereby an ongoing research project evolves through interactions with the research literature.

## Introduction

An important part of the research process is literature search: identifying prior work that is of relevance to the present idea being investigated. In many cases, this is an activity that a researcher may defer until writing up the results of the project, in which case, it is primarily an activity one does because one “has to” rather than an activity that can substantially change the course of the research. In some cases, whether due to negligence or the difficulty of finding related works, a researcher may never come across the fact that someone had previously tackled the same problem or made a similar discovery, and perhaps only years later (if ever) it may be realized (Merton, 1963; Ke et al., 2015; Sacks, 2002). But at its best, this is an activity that leads to new insights into the research problem, generates new ideas, and alters the course of the research. In fact, in some cases, searching for related work can *become* the research process itself; through connecting various pieces of research literature alone, one can discover previously undiscovered public knowledge (Swanson, 1986).

Despite the importance of prior literature in the research process, there has been little effort, if any, dedicated to developing a typology of related works, that is, a typology of relationships that might exist among different pieces of research literature. (Of course, it is entirely possible that such a typology has been constructed, but I have missed it due to an inadequate search of the literature!) In this paper, I propose such a typology to help us better understand the kinds of prior work that might have bearing on a research project. I first present a form of knowledge representation that can theoretically be used to represent any piece of research literature or research project. I then present a typology of relationships that can connect two pieces of research in terms of operations that can apply to the two representations, thereby resulting in a representation of the relationship. I will demonstrate the various operations and how they might be employed with a variety of examples from different fields, including medicine, physics, psychology, history and philosophy of science, machine learning, education, and neuroscience. The same form of representation applies to both published research literature and research projects or topics, whether nascent or fully-fledged. In fact, some of the relationships discussed below make more sense in the context of research projects (or broader research agendas) that can dynamically evolve as relevant literature is encountered, rather than research papers whose underlying representations are static. As such, I will use the terms publication, literature, project, and topic somewhat interchangeably.

The specific representation I use is borrowed from structure-mapping theory (Gentner, 1983; Falkenhainer et al., 1989), which was originally developed as a way to structurally represent analogies. Structure-mapping theory is particularly useful here, both because we can use it when discussing abstractions and analogies, and because the underlying representation can also handle other types of relationships among literature. I could have instead used other forms of knowledge representation, such as conceptual graphs (Sowa, 1976), entailment meshes (Pask et al., 1975; Pask, 1988), or category theoretic representations like ologs (Spivak & Kent, 2012). There may be relative advantages to each of these, but the representation used here is both simple and powerful enough to clearly demonstrate the typology. The exact choice of representation may need further consideration if one wants to perform inference on the representations or utilize them in information retrieval tools. For now, we are not concerned with how one might construct these representations or even the fidelity with which it is possible (though we revisit these questions in Sect. 6). The possibility that research projects could *in theory* be represented in the way described below is sufficient to formulate the typology.

While the form of representation and typology presented below may not be directly used in information retrieval tools, I contend that they may be useful in guiding the overall direction that research on such tools might take (e.g., what kinds of papers should a tool search for?). Moreover, the typology may provide some clarity to researchers going through the literature search process for a project. Constructing a graphical representation of one’s paper may be a useful exercise, and can possibly illuminate different searches that are needed to find related work. Seeing how the representation of one’s paper changes over time can also be a useful documentation of the research and literature search process. Beyond such potential practical uses of the typology, I believe it can simply be beneficial to understand the various ways in which one product of research may relate to another. If alongside the physical and social worlds, the world of research literature “also qualifies as an endless frontier” (Swanson, 1986, p. 115), then our efforts to make sense of the former should be accompanied by efforts to make sense of the latter.

## Related work on related work

Related work on related work exists in a number of different disciplines. Literature search is central to all research after all! Fittingly, the typology we develop combines research that exists in different, largely isolated, strands.

In the information sciences and medicine, work on “literature-based discovery” (LBD), dating back to Swanson (1986), is concerned with making new scientific discoveries by establishing novel connections between different pieces of literature. Swanson (1986) describes literature-based discovery as a form of scientific discovery that takes place in Karl Popper’s world 3—the “world of the products of the human mind” (Popper, 1978, p. 144)—whereby search functions are likened to scientific theories and the “logic of undiscovered public knowledge” (p. 116) is analogous to the logic of scientific discovery. In doing so, Swanson (1986) made a contribution to the philosophy of science, though it seems to have not been recognized in the philosophy of science community. A number of different information-retrieval techniques have been proposed to aid in LBD (Smalheiser, 2017; Sebastian et al., 2017). Some authors have presented categorizations of different types of “undiscovered public knowledge” or different forms of LBD (Davies, 1989; Smalheiser, 2017). While these categorizations can be useful in aiding researchers who want to perform literature-based discovery, our typology has a somewhat broader scope in that not all related work necessarily results in LBD. LBD is one potential use case of literature search, and its various methods span across the relationships in the typology presented here, as discussed below.

More broadly, in information retrieval, the notion of “relevance” is central, and some researchers have tried to develop theories around what relevance is—typically conceived of as the relationship between an information need and a document (Saracevic, 1975, 2016; Huang & Soergel, 2013). Green (1995) and Huang and Soergel (2013) pointed out that most discussions of relevance are around “topic matching,” but that this is only one form of relevance. Green and Bean (1995) then constructed a typology of different notions of relevance, and Huang (2009) expanded this to a typology consisting of over 200 notions of relevance. Huang (2009) considers three broad categories of relationships: (1) “What functional role a piece of information plays in the overall structure of a topic,” (2) “How information contributes to users’ reasoning about a topic,” and (3) “How information connects to a topic semantically” (p. 411). As examples of functional roles, an information source might present a solution to a problem, the cause of an effect, etc. As examples of contributing to reasoning, an information source might provide an analogy to the topic or might be used to deduce something about the topic. While this work is very relevant to the present paper, there are two key differences. First, their work is about the broader concept of relevance between information and needs, while this paper focuses on relevance in academic literature. One would expect that many of the kinds of broader relevance typologies would also hold for research publications, but given the particularities of literature search and the role it plays in the broader process of scientific research, it seems worth studying in its own right. Second, these prior typologies largely focus on the variety of *semantic* relationships between two topics, while the approach we present here views relevance in terms of *operations* that operate on knowledge representations of topics. In this sense, the typology I present here can express how to relate different research topics in terms of a small number of mathematically precise operations (that are hopefully easy to remember), rather than a plethora of different possible semantic relationships. The two approaches are complementary, but I contend that the approach taken here is more useful for conceptualizing the evolution of a research project over time.

In computer science and artificial intelligence, there has been a recent thread of work on citation recommendation, concerned with identifying relevant citations given a piece of text and possibly other meta-data (e.g., authors, etc.) (Strohman et al., 2007; Liang et al., 2011; Ren et al., 2014; Bhagavatula et al., 2018). Interestingly, this work has not really considered automated techniques for LBD, and it does not cite the vast literature on LBD or on relevance. Indeed, most of the work in this area is concerned with topic matching (finding citations that topically overlap). One notable exception is work by Chan et al. (2018) and Kang et al. (2022). Chan et al. (2018) presented a technique that combines crowdsourcing and machine learning to find analogies between different papers. They utilize a “soft” relational schema, a very coarse-grained representation of a research paper; they explicitly avoid using representations like the one described below, because they can be very difficult to construct for many publications. Kang et al. (2022) built on this work by training deep learning algorithms on the crowdsourced representations of abstracts to be able to automatically detect the “purpose” and “mechanism” of a paper. An analogy in this context is two papers that have a similar underlying purpose but achieve that purpose through a different mechanism. Kang et al. (2022) used this to prototype an analogical search engine for scientific literature. While their representation may be useful for LBD, I contend that it can only capture certain kinds of relationships between papers, and, as I discuss further below, some of their methods do not appear to actually look for analogies as per the typology we develop below. As such, our typology can potentially be useful in classifying the different kinds of relationships that various existing LBD and citation recommendation methods can uncover, and the kinds of relationships that they cannot.

## A representation of a research project

In our representation, a research project or publication $P \in \Pi$ is represented as a set of entities and relations, $P = (E, \mathcal {R})$. An entity conceptually represents any specific topic of relevance to the project, usually expressed as a noun or a noun phrase (e.g., DNA, the civil rights movement, high blood pressure, theorems). Notice that entities can come in different degrees of specificity (e.g., theorems vs. Gödel’s first incompleteness theorem); the important thing is that entities across all topics and publications are represented at the same level of granularity. We allow entities to be hierarchically defined as functions of other entities (e.g., the entity “volume of a cup” can be thought of as the “volume of” function applied to “cup”).

Relations define a relationship between some number of entities, such that the predicate $R(e_1, e_2, \dots , e_n)$ indicates that $e_1$, $e_2$, ..., $e_n$ are related as specified by the relation *R*. Binary relations are perhaps the most common. For example, in the sentence “stress causes high blood pressure”, “causes” is a relation that takes relates two entities (in this case, “stress” and “high blood pressure”). We might represent this as causes(stress, high blood pressure). As an example of a tertiary relation, consider the sentences “ribosomes translate mRNA into sequences of amino acids” and “Arab translators translated Greek texts into Arabic translations”; they could both be said to use the relation x-translates-y-into-z (though if we think the word “translates” has a very different semantic meaning in these two cases, we could suggest there are two different relations at play here). We also allow for unary relations; for example, “blood pressure is high” can be represented as is-high(blood pressure). Unary relations are called attributes in structure-mapping theory and they effectively allow assigning adjectives to entities; for example, high(blood pressure) would mean “high blood pressure.” With slight abuse of notation, I will use unary relations both as relations (e.g., is-high(blood pressure)) and as attributes (e.g., high(blood pressure)). Finally, we allow for higher-order relations, which take relations as input instead of, or in addition to, entities. causes is a higher-order relation because we can say, for example, causes(provided(treatment(subjects), New Curriculum), learn-more-than(treatment(subjects), control(subjects))).

As with Gentner’s (1983) structure-mapping theory, the classification of relationships between research has more to do with the structure of the representation (i.e., the presence of certain entities and relations) rather than the semantic meaning of the nodes. However, semantics still play an important role in informing whether a particular relationship is sensible or important in a particular situation. That is, someone without a semantic understanding of a given domain can still apply the operators described below in the sense that one can execute $4 + 7$ and $4 \times 7$, without regard to which operation makes more sense in the given situation. Furthermore, one aspect of semantics is necessary in the application of some of the operators. Namely, there is a general relation, “is a” (or “is an instance of”), which can capture any situation where a particular entity can be categorized as a special case or instance of another entity. Consistent with earlier work on knowledge representation, we will refer to this relation as is-a (Brachman, 1983). For example, is-a(Gödel’s first incompleteness theorem, theorem) and is-a(the civil rights movement, historical occurrence). A single entity can be an instance of many entities (e.g., a cat can be considered an animal, a pet, and an Internet phenomenon). The is-a operator is also reflexive (e.g., is-a(cat, cat)). Finally, with slight abuse of notation, we will also have is-a be a higher-order relation that can designate when one relation is an instance of another. For example, is-a(holds(person, ball), possesses(person, object)), because holding something is a special case of possessing it and a ball is an object. Some of the operators below can only be applied with an understanding of what things are instances of other things; however, when the relationship is more abstract, sometimes even a domain expert will not readily see these connections.

The set of entities and relations that are used in the representation of a research publication will likely not include all entities and relations included in that publication (e.g., all nouns and verbs), but rather they should include the concepts that are focal to that publication. Of course, that is somewhat subjective, but a useful heuristic is to include all entities and relations that are involved in a system of relationships that might be worth providing citations in reference to, as well as any new entities and relations that are being introduced in the paper. For example, in a paper that runs an experiment with seven conditions, the number of seven is probably not an entity that should be included, but in Miller’s (1956) paper on working memory capacity or a paper on the religious symbolism of the number seven, it likely should be included.

As suggested above, there is no single correct way to represent a research project. In fact, there can be multiple different *views* of a research project, which induce different representations. Each of these views can be more or less useful depending on how they are to be used. Moreover, even simple relations can be expressed in different ways. For our purposes, there is a relationship between two research projects if there is at least *some* view of each that permits the relationship. Since we are not concerned with the practical side of how to best represent projects here, we do not worry about how one would go about discovering the “right” views. In practice though, seeing two related papers from the “wrong” viewpoint is one reason why researchers and information retrieval tools might not notice an important relationship.

**Fig. 1:**
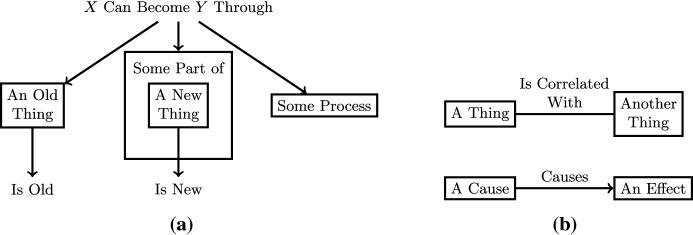
Examples of how to graphically represent research projects/publications. **a** An example of a tertiary relation with three entities that would be read as “An old thing can become some part of a new thing through some process.” There are also two unary relations: is-old and is-new. **b** Two examples of binary relations. The causes relation is asymmetric while the correlated relation is symmetric

We can represent these representations graphically using a graph-like structure as shown in Fig. 1a. Boxes indicate entities, and the text outside of boxes indicate relations. The arrows coming out of a relation point to its arguments in order from left to right. Nested boxes (e.g., “some part of a new thing”) show hierarchically defined entities. For simplicity, we show binary relations as labeled directed edges for asymmetric relations and labeled undirected edges for symmetric relations, as shown in Fig. 1b.

This representation could be couched in the language of model-theoretic philosophy of science (Suppes, 1957, 1960), in particular using the partial structures formalism (French, 2000; Da Costa & French, 1990), which is also often expressed in terms of entities and (partial) relations. Doing so may be appealing since it would connect literature search to an existing framework for discussing scientific theories. The partial structures formalism has also been used in describing analogies and abstractions in science and provides a way to formalize research undergoing change. However, the ideas presented here not only apply to formal scientific theories, but also to non-scientific literature and more nascent representations of scientific topics, and I do not want to associate the typology presented here with a particular interpretation of scientific theories.

## A typology of related works

We can now describe the different kinds of relationships that can exist between a research project and prior work. Suppose we have a research project $P = (E_P, \mathcal {R}_P)$ and a piece of literature $L = (E_L, \mathcal {R}_L)$. We assume that *P* is an ongoing project that can potentially change, while *L* is already published literature and hence static. Below we describe a set of operations that can be used to describe the relationship between *P* and *L*. These operations are functions that take the representations of *P* and *L* as inputs and output a representation $\rho$ of the relationship between *P* and *L* (as defined by the operation). Since we allow for composing these operations in sequence, some of the operations will actually take as input *P*, *L*, *and* our current representation of the relationship between the two ($\rho _i$), and will output a modified representation of the relationship ($\rho _{i+1}$). Moreover, when applying multiple operations in sequence, we may want to keep track of the ongoing relationship, which we can do by merging multiple relationships (i.e., taking the union of entities and the union of relations in the sequence of relationships). After each operation is applied, we can also potentially modify *P*^1^, thereby modifying the relationship between *P* and *L* as well. The series of operations and modifications reflects the iterative and influential nature of literature search in the research process. The operations, described below, are called intersection, interpretation, expansion, abstraction, reification, analogy, and substitution. Table 1 lists some basic information about the operations, which may be useful when reading the sections below. I do not make any claims that the typology presented here is complete. There might be other operations, or perhaps more useful categorizations of the operations presented here, which can be elucidated upon in future work. In what follows, I will describe each of the operators in words as well as mathematical formalism when needed; readers can safely skip the mathematical formalism and still grasp the key ideas.

**Table 1:** A list of the operations in the typology along with their inputs and outputs

Operation	Verbal description	Inputs	Output
Intersection	*P* intersects with *L*	*P*, *L*	$(E_{PL}, \mathcal {R}_{PL})$
Interpretation	*P* interprets *L*	*P*, *L*, $\rho _i$	$(E_{\rho _{i}} \cup E_{PS}, \mathcal {R}_{\rho _{i}} \cup \mathcal {R}_{PS})$
Expansion	*P* is expanded upon by *L*	*P*, *L*, $\rho _i$	$(E_{\rho _{i}} \cup E_{LS}, \mathcal {R}_{\rho _{i}} \cup \mathcal {R}_{LS})$
Abstraction	(some aspect of) *P* is abstracted by *L*	*P*, *L*	$(E_{PS} \cup E_{LS}, \mathcal {R}_{PS} \cup \mathcal {R}_{LS} \cup \textsc {is-}a)$
Reification	(some aspect of) *P* is reified by *L*	*P*, *L*	$(E_{PS} \cup E_{LS}, \mathcal {R}_{PS} \cup \mathcal {R}_{LS} \cup \textsc {is-}a)$
Analogy	(some aspect of) *P* is analogous to *L*	*P*, *L*	$(E_{PS} \cup E_{LS}, \mathcal {R}_{PS} \cup \mathcal {R}_{LS} \cup \textsc {analogous})$
Substitution	(some aspect of) *P* can be substituted by *L*	*P*, *L*	$(E_{PS} \cup E_{LS}, \mathcal {R}_{PS} \cup \mathcal {R}_{LS} \cup \textsc {substitutes})$

For the meaning of the terms used in the Output column, see the appropriate section

### Intersection

The first and probably most prevalent operation is **intersection**, which outputs a subset of entities that are shared by *P* and *L* and a subset of relations shared by the two. Specifically, intersection outputs a representation $\rho = (E_{PL}, \mathcal {R}_{PL})$, where $E_{PL} \subseteq E_P \cap E_L$ and $\mathcal {R}_{PL} \subseteq \mathcal {R}_P \cap \mathcal {R}_L$. The exact subset depends on what is determined to be relevant between the two representations. A simple special case of this would be when *P* and *L* share just a single entity. For example, suppose *P* and *L* both have to do with DNA, but one is about DNA to solve computational problems (Adleman, 1994) and the other is about DNA vaccines for coronavirus (Callaway, 2020). It is unlikely that these publications have other entities in common. In many such cases, publications are not worth citing, and such an intersection would actually not be relevant. A relationship is worth noting when the degree of overlap is large enough; this can be measured by associating some degree of importance to each entity in *P* and taking the sum (or some non-linear function) of importances across all the entities in $\rho$.

In some cases, overlap in a single entity may be enough to warrant citation or even to alter the course of a research project. For example, one of the examples that Swanson (1986) gives for undiscovered public knowledge has to do with a potential research publication on the “all swans are white hypothesis,” a hypothesis that states that all swans are white. This hypothesis could be supported inductively if there was a lack of any documented evidence of black swans. As Swanson (1986) says:

Suppose for the sake of argument that scientists living in a remote part of the world were to publish, in a local wildlife journal, some observations about a family of black swans living on a nearby lake. We suppose further that the report comes from a half-dozen people who are reliable observers, and that they are unaware that other people in the world think that all swans are white. (p. 109)

**Fig. 2:**
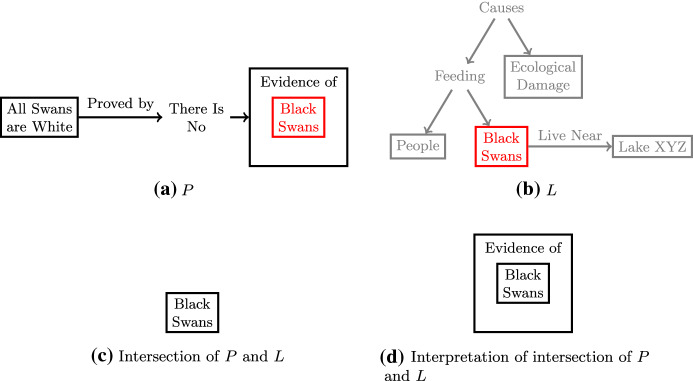
Swanson’s (1986) black swans example as an example of intersection

As shown in Fig. 2, the potential all-swans-are-white hypothesis publication (*P*) is represented using three entities and two relations, although it can be interpreted as two entities and the relationship between them (“the all-swans-are-white hypothesis is proved by the fact that there is no evidence of black swans”); on the other hand, the article in the wildlife journal (*L*) only concerns itself with black swans and possibly other topics of local interest. As such, the two articles overlap in only one entity: black swans. It just so happens that the existence of black swans is a critical refutation of the theory (i.e., “evidence of black swans” is a very important entity in *P*), and so this single article can change the course of the research project (e.g., the authors publish a refutation of the all-swans-are-white hypothesis rather than a proclamation of it).

Notice that the intersection of the two articles was “black swans” not “evidence of black swans.” (The wildlife journal is not trying to present evidence of black swans; it is discussing a piece of wildlife whose existence they never called into question.) The intersection of “black swans” by itself is not necessarily meaningful. Another paper that discusses black swans but provides no evidence for them is of less value to *P*. How then can we capture the obvious fact that *L* presents evidence of black swans, even though it is not captured in its representation? The answer lies in the interpretation operation.

### Interpretation

An **interpretation** takes an existing relationship between *P* and *L* and adds additional entities and/or relations from *P* (not included in *L*) that can help interpret the current relationship. Namely, if $\rho _{i} = (E_{\rho _{i}}, \mathcal {R}_{\rho _{i}})$ is the output of a previous operation, then $\rho _{i+1} = (E_{\rho _{i}} \cup E_{PS}, \mathcal {R}_{\rho _{i}} \cup \mathcal {R}_{PS})$, where $E_{PS} \subseteq E_P \setminus E_L$ and $\mathcal {R}_{PS} \subseteq \mathcal {R}_P \setminus \mathcal {R}_L$. (I use *PS* and *LS* as subscripts to denote subsets of *P* and *L*.) A natural use of interpretation is to apply it after an intersection. For example, in the black swans example above, we can interpret the intersection of *P* and *L* as being “evidence of black swans.” Clearly, *L* does present evidence of black swans, but it was not interpreted that way until it was interpreted in light of *P*. Notice that if a researcher conducting project *P* were to construct the representation of *L*, they might do so according to their interpretation, whereby “evidence of black swans” would appear in *L*. Therefore, interpretation steps may often be implicit or hidden in the particular view of *L* that a researcher adopts. In this paper, I *try to* represent prior work in a way that is faithful to the original authors’ meaning, though we must recognize that views of prior work will always be informed by our worldview.

### Expansion

An **expansion** takes an existing relationship between *P* and *L* and adds additional entities and/or relations from *L* (not included in *P*) to potentially expand the content of *P* or to bring new insights into the picture. Notice that structurally, the expansion operation is equivalent to the interpretation operation with *P* and *L* swapped; however, semantically, the two are often quite different. An expansion will often result in a change in *P*. As a result, it makes the most sense when *P* is an ongoing research topic (or a follow-up investigation to published work), rather than a final publication. Once *P* has changed to $P'$ to incorporate the new entities and relations, what was once an expansion between *P* and *L* may be viewed as an intersection between $P'$ and *L*. Therefore expansions play developmental roles in the research process, which are often not captured in publications. That is, many research projects may have changed course as a result of particular publications, but the final publication may only refer to the relationship to prior work at the time of publication, rather than the developmental influence of that prior work.

For example, in the related works section above, I acknowledged connections to Chan et al. (2018); these connections would be viewed as intersections (e.g., both papers have to do with academic literature, analogies, knowledge representation, etc.). However, what I did not state was that reading Chan et al. (2018) led me to read about structure-mapping theory (Gentner, 1983), and the two publications combined (and considered in relation to Swanson (1986)) resulted in the beginnings of this paper. That is, before this paper was even conceived of, the aforementioned prior works resulted in a series of expansions, which turned into the present piece only after many iterations, which involved a series of other operations applied to various publications (some of which are cited, and some of which may not be). This reflects the role of literature search in the messy process that is research. I suspect that researchers rarely document the series of expansions (and other steps) that lead to the final state of a publication.

In fact, at times, some prior work may only play the role of a stepping stone to discovering other, more relevant, prior works. That is, an expansion of *P* by $L_1$ may result in an exploration of the new entities in the expansion, which results in discovering $L_2$, which intersects with *P*. At that point, $L_1$ may no longer really be relevant; that is, the extent of $L_1$’s relevance may be better captured by $L_2$.

One broad category of expansions falls under Swanson’s (1986) second example—“A Missing Link in the Logic of Discovery” or what is often referred to as the ABC model. As Swanson (1986) originally expressed it:

Suppose the following two reports are published separately and independently, the authors of each report being unaware of the other report: (i) a report that process A causes the result B, and (ii) a separate report that B causes the result C. It follows of course that A leads to, causes, or implies C. That is, the proposition that A causes C objectively exists, at least as a hypothesis. (p. 110)

**Fig. 3:**
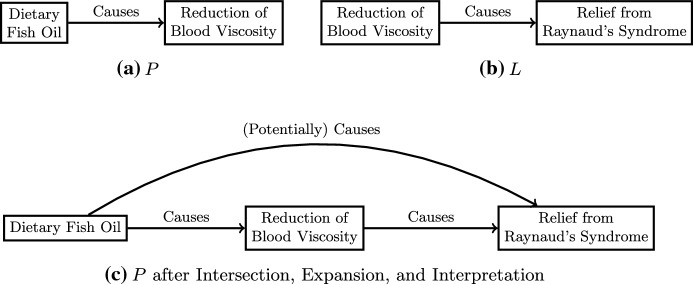
Swanson’s (1986) example of the ABC model as an example of expansion

Swanson gave a specific example of a discovery he made (the first of his several literature-based discoveries in medicine): connecting (a) literature on how fish oil causes a reduction of blood viscosity with (b) literature on how reducing blood viscosity leads to an improvement in symptoms of Raynaud’s syndrome. The intersection of these two literatures is the entity “reduction of blood viscosity.” An expansion adds the causal link to “relief from Raynaud’s syndrome” and that link is then interpreted in light of the connection to “dietary fish oil.” Connecting these two literatures via these steps can result in a change in *P* as shown in Fig. 3. Notice that the addition of a new causal relation between dietary fish oil and relief from Raynaud’s syndrome was inferred from this expansion, but had never been experimentally shown or even published about. Two years later, a clinical trial independently confirmed this hypothesis (Swanson & Smalheiser, 1996).

Literature-based discovery often involves this kind of linking between two “non-interactive literatures,” literatures that are rarely, if ever, cited in the same publications (Swanson & Smalheiser, 1996). However, expansion need not always be between two non-interactive literatures. Indeed, researchers may often be unaware of highly relevant work within their own research community (or other interactive literatures) that build upon the concepts they are investigating. Such cases can often be caught by the researchers themselves when conducting a more expansive literature review, or by reviewers during the peer review process, but likely often go undetected.

### Abstraction

An **abstraction** applies if *P* contains a subset of entities and relations that are instances of entities and relations in *L*. In other words, we have an abstraction when *L* contains a more abstract or generalized representation of part of *P*. An abstraction can still consist of concrete entities and relations as long as they are more general or more abstract than the entities and relations in *P* (e.g., as suggested above is-a(cat, animal), is-a(cat, Internet phenomenon), and is-a(the civil rights movement, historical occurrence) can all be single entity abstractions).

Describing an abstraction mathematically requires a bit more care than for previous operations since abstractions must be semantically “consistent” across the entities and relations involved. Formally, an abstraction applies if there is a subset of entities and relations in *P*—say $E_{PS} \subseteq E_P$ and $\mathcal {R}_{PS} \subseteq \mathcal {R}_P$—and a subset of entities and relations in *L*—say $E_{LS} \subseteq E_L$ and $\mathcal {R}_{LS} \subseteq \mathcal {R}_L$—such that the following four conditions hold:

1. For all $e \in E_{PS}$, there exists a $\tilde{e} \in E_{LS}$ such that *e* is an instance of $\tilde{e}$.
2. For all $R \in \mathcal {R}_{PS}$, there exists a $\tilde{R} \in \mathcal {R}_{LS}$ such that *R* is an instance of $\tilde{R}$.
3. For all $R \in \mathcal {R}_{PS}$, if $R(e_1, e_2, \dots , e_n)$, then $\tilde{R}(\tilde{e}_1, \tilde{e}_2, \dots , \tilde{e}_n)$, where $R, e_1, \dots , e_n$ are instances of $\tilde{R}, \tilde{e},_1 \dots , \tilde{e}_n$ respectively.
4. At least some $e \not = \tilde{e}$ or some $R \not = \tilde{R}$.

The last condition is required to make sure the abstraction is not simply mapping identical representations (in which case it would just be an intersection). The resulting representation is $\rho = (E_{PS} \cup E_{LS}, \mathcal {R}_{PS} \cup \mathcal {R}_{LS} \cup \textsc {is-a})$, where $\textsc {is-a}(e, \tilde{e})$ and $\textsc {is-a}(R(e_1, e_2, \dots , e_n), \tilde{R}(\tilde{e}_1, \tilde{e}_2, \dots , \tilde{e}_n))$, for all *e*, $\tilde{e}$, *R*, and $\tilde{R}$ as defined in the conditions above.

**Fig. 4:**
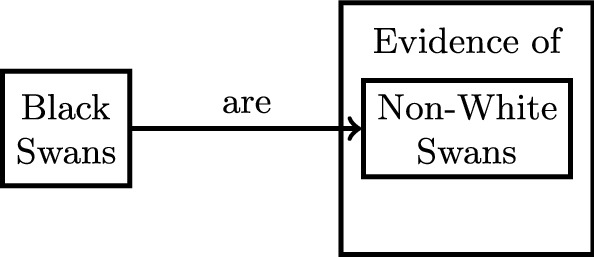
The black swans example revisited. The relationship between *P* and *L* is now an interpretation of an abstraction of *L*. Notice that we used “are” instead of “is a” simply because the entities are expressed in plural

Abstractions need not be profound. Consider the black swans example again. The way I presented it above was actually a bit disingenuous: black swans are not the only evidence that disproves the all-swans-are-white hypothesis; any non-white swans would. Thus it might be more accurate to replace the “black swans” entity with “non-white swans” in Fig. 2a. The relationship between *P* and *L* then first involves an abstraction (instead of an intersection)—namely is-a(black swans, non-white swans)—followed by an interpretation, as shown in Fig. 4. This is a rather trivial kind of abstraction, which likely happens all the time when interpreting prior work in the context of current work.

A more substantial form of abstraction is whenever *P* reports on empirical findings that can be subsumed into an existing theory described by *L*. For example, if researchers find that students in a collaborative problem-solving activity learned more than students who were working on the activity on their own, then they might see the ICAP hypothesis (Chi & Wylie, 2014), which posits that interactive learning is better than constructive learning, as an abstraction.

Finally, perhaps the most interesting (but also rarest) form of abstraction is when a body of research is interpreted or a problem is solved using some abstract formalism or framework that exists in the literature (often in a different field). For example, a notable example in the history of science is the introduction of group theory to quantum mechanics to solve certain problems related to symmetry (French, 2000; Scholz, 2006). According to French (2000):

the relationship between mathematics and physics is represented in terms of an embedding of a scientific theory into a mathematical structure. This effectively gives the theory access to ‘surplus’ mathematical structure which can play an essential role in the further development of theory. (p. 104)

This “surplus structure”—a term originally from Redhead (1975)—is represented in our typology by expansion steps that can follow the abstraction. Namely, once a connection is made between *L* (say group theory) and *P* (a particular problem in physics), an expansion can be applied to bring new mathematical machinery from *L* to bear on *P*. Furthermore, an interpretation of the abstraction of *L* in light of *P* might result in new insights that could lead to further developments in *L* (if we do not consider *L* to be static literature). As French (2000) states, “it is important to acknowledge that both group theory and quantum mechanics were in a state of flux at the time they were brought into contact and both subsequently underwent further development” (p. 110).

### Reification

A **reification** is the inverse of an abstraction. That is, a reification has the same definition of an abstraction, except that *P* and *L* are exchanged. We can say *P* is reified by *L* if *L* is abstracted by *P*. A reification can occur when prior work might contain a concrete example of a phenomenon, which one’s present work presents in more abstract or general terms. Reifications will often be used when interpreting prior empirical findings in light of a new theoretical framework. For example, when articulating his theory of the structure of scientific revolutions, Kuhn (2012) drew on myriad concrete historical examples from the history of physics, astronomy, chemistry, and other fields. These findings are reifications of particular components of Kuhn’s theory (e.g., paradigms, anomalies, paradigm shifts, etc.).

**Fig. 5:**
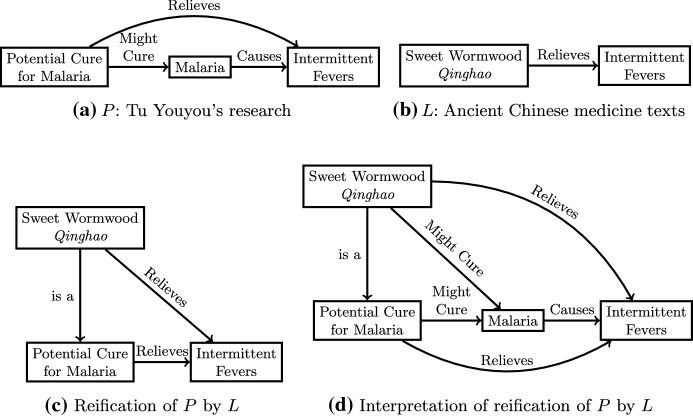
The discovery of sweet wormwood as a cure for malaria as an example of reification

A reification can also make sense when one is in a formative stage of a project where some of the specifics have not yet been determined. For example, consider Tu Youyou’s work on finding a cure for malaria in the 1970s for which she won the Nobel Prize in 2015. The problem that Tu and her team were working on is represented in Fig. 5a. According to Tu (2015):

After thoroughly reviewing the traditional Chinese medical literature and folk recipes and interviewing experienced Chinese medical practitioners, I collected over two thousand herbal, animal and mineral prescriptions within three months after initiation of the project.

One of the substances that showed some initial promise was sweet wormwood (*qinghao*), which was shown in the literature to cure intermittent fevers, as shown in Fig. 5b. Therefore sweet wormwood is a reification of a potential cure for malaria, as shown in Fig. 5c, and this can be interpreted in the broader research of finding a cure for malaria, as shown in Fig. 5d. Yu went on to identify artemisinin as an actual cure for malaria, but there was an additional step of literature-based discovery needed first, which we will return to later.

### Analogy

An **analogy** applies when *P* and *L* both have a subset of entities and relations that have a shared abstraction. More formally, using the same notation as above, an analogy applies if there exists some other representation $A = (E_A, \mathcal {R}_A)$ (representing an abstraction) and the following four conditions hold^2^:

1. For all $\tilde{e} \in E_A$, there exists an $e \in E_{PS}$ and an $e' \in E_{LS}$ such that *e* and $e'$ are both instances of $\tilde{e}$.
2. For all $\tilde{R} \in \mathcal {R}_A$, there exists an $R \in \mathcal {R}_{PS}$ and an $R' \in \mathcal {R}_{LS}$ such that *R* and $R'$ are both instances of $\tilde{R}$.
3. For all $\tilde{R} \in \mathcal {R}_{A}$ and for every pair $R \in \mathcal {R}_{PS}$ and $R' \in \mathcal {R}_{LS}$ such that *R* and $R'$ are both instances of $\tilde{R}$, if $\tilde{R}(\tilde{e}_1, \tilde{e}_2, \dots , \tilde{e}_n)$ then $R(e_1, e_2, \dots , e_n)$ and $R'(e'_1, e'_2, \dots , e'_n)$, where $e_i$ and $e'_i$ are instances of $\tilde{e}_i$ for all *i* and *R* and $R'$ are instances of $\tilde{R}$.
4. At least some $e \not = e'$ or some $R \not = R'$.

We say that $\textsc {analogous}(e,e')$ if and only if condition 1 holds for *e* and $e'$ and similarly we say that $\textsc {analogous}(R(e_1, e_2, \dots , e_n), R'(e'_1, e'_2, \dots , e'_n))$ if and only if the conditions 2 and 3 above hold for those entities and relations. The representation that results from an analogy operation is $\rho = (E_{PS} \cup E_{LS}, \mathcal {R}_{PS} \cup \mathcal {R}_{LS} \cup \textsc {analogous})$.

Analogies can span from shallow analogies between two instances of a similar phenomenon in the same field to deep analogies across scientific fields that share little apparent relation to one another on the surface. The further removed that *P* and *L* are from the abstraction *A*, the deeper the analogy becomes (and typically, the harder to notice). Concretely identifying the abstraction implicit in an analogy is not necessary, and in some cases, it can actually be difficult to do, but I suggest that doing so may be a useful exercise (and could lead to refining the analogy).

Like expansions, analogies can sometimes result in modifying *P* by looking at the research project in a whole new light. Like expansions, this also means the way in which an analogy might have helped develop *P* over time may not always be apparent from the final product. Even if a publication discusses an analogy, it may not always be clear if that analogy was instrumental in developing the idea in the first place or if it was an afterthought that the two ideas were related.

An example of an analogy where the impact of prior work on a research project *is* actually made explicit is the analogy between Thomas Kuhn’s historical philosophy of science and Jean Piaget’s psychological and epistemological theory of how a child develops knowledge. In *The Structure of Scientific Revolutions*, Kuhn (2012) gives us a brief sense of his indebtedness to Piaget:

A footnote encountered by chance led me to the experiments by which Jean Piaget has illuminated both the various worlds of the growing child and the process of transition from one to the next. (p. xi)

The extent of this has recently been clarified by historians examining Kuhn’s other works and archival materials (Galison, 2016; Burman, 2020). For example, Kuhn (1977, as cited in Burman, 2020) states:

Almost twenty years ago I first discovered, very nearly at the same time, both the intellectual interest of the history of science and the psychological studies of Jean Piaget. Ever since that time the two have interacted closely in my mind and in my work. (p. 21)

**Fig. 6:**
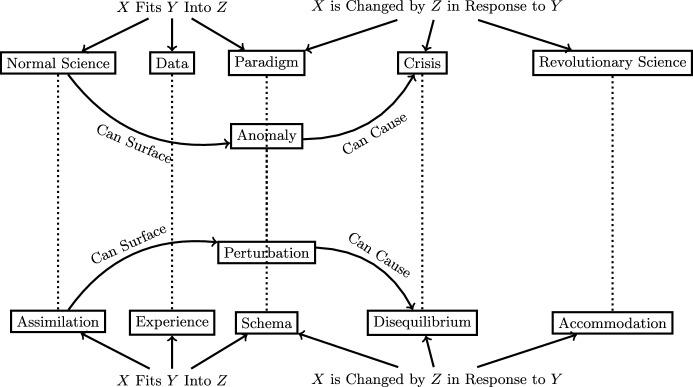
The representation of the analogy between Kuhn’s *The Structure of Scientific Revolutions* and Piagetian theory. The analogous relations are shown as dotted lines without labels for ease of reading

So what was the nature of this close interaction? One can draw a clear analogy between the two. At risk of oversimplification, a representation of the analogy between Kuhn’s theory and Piaget’s is shown in Fig. 6, adapted from a mapping given by MacIsaac (1991). This is not at all to say that this is the precise analogy that Kuhn drew which led to a refinement of his theory as presented in *The Structure of Scientific Revolutions*. However, he probably made similar mappings that changed over time as he developed his theory. Similar analogies can also be drawn from Kuhn’s theory to gestalt theory and Bruner and Postman’s (1949) psychological theory of how people perceive incongruities, both of which Kuhn (2012) explicitly builds off of. Interestingly enough, the Piagetian analogy, while very influential on the development of Kuhn’s theory, was not retained in the final representation of his book, while the analogies to gestalt theory and Bruner and Postman (1949) were explicitly an important part of his narrative. Note that the relations in *P* and *L* are identical in this case, but this need not be the case in general; in fact, they may only be identical because I constructed them that way, but perhaps if the representations were to be derived independently, the relations would be non-identical, but share a common abstraction.

To provide a more recent example of analogy, we can consider the relationship between the recent machine learning literature on fairness (*P*) in relation to older literature from the 1960s-1970s on fairness in educational and employment testing (*L*). As Hutchinson and Mitchell (2019) point out, the two literatures share much in common including many mathematical definitions of fairness. To formalize this, Hutchinson and Mitchell (2019) explicitly construct an analogy between the two literatures:

Test items (questions) are analogous to model features, and item responses analogous to specific activations of those features. Scoring a test is typically a simple linear model which produces a (possibly weighted) sum of the item scores....Because of this correspondence, much of the math is directly comparable; and many of the underlying ideas in earlier fairness work trivially map on to modern day ML fairness. “History doesn’t repeat itself, but it often rhymes”; and by hearing this rhyme, we hope to gain insight into the future of ML fairness. (p. 49)

Their last sentence suggests that the goal of pointing out the relationship between these two literatures are further steps of expansion and interpretation, or in other words, exploiting the “surplus of structure.” Indeed, the authors surface several definitions from test fairness that had not been proposed in machine learning (i.e., an expansion). Notice that in this case, the underlying abstraction may not be immediately obvious (e.g., what is the abstraction underlying both a test item and a feature?); in fact, in some cases, there may not be a simple word or phrase to describe the abstraction, but the fact that a clear analogy can be drawn indicates that there must be some more abstract underlying representation.

Finally, in my own research, I have found that there is an analogy between debates in education research and the bias-variance tradeoff in machine learning (Doroudi, 2020). Here an analogy was determined by directly formulating the abstraction (a generalized version of the bias-variance decomposition theorem). This abstraction has four components that any instance must specify: a target, an approximator, a random mechanism, and a source of randomness; once these components are specified, one can derive other phenomena (e.g., the meaning of bias, variance, etc.). This naturally sounds very abstract, but it is more concrete once instantiated in specific contexts. Table 2 gives an example of the analogy between these concepts in machine learning and debates around pedagogy. Once this analogy is drawn, it may be possible to expand techniques that are developed in machine learning to bear on educational debates (Doroudi, 2020). One benefit of making the abstraction concrete is that the same abstraction can be used to draw analogies to other fields as well.

**Table 2:** A mapping of the bias-variance decomposition from machine learning (*L*) to its analog in the study of pedagogy (*P*), along with the abstraction that connects the two (*A*)

Abstraction (*A*)	Machine learning (*L*)	Pedagogy (*P*)
Target *T*	Function *f*	Optimal educational experience
Approximator $\hat{T}$	Estimator $\hat{f}$	Actual educational experience
Mechanism $\mathcal {M}$	Machine learning algorithm	Instructional intervention
Source of randomness	Dataset $D \sim \mathcal {P}_D$	Stochasticity in instructional intervention
High bias / low var $\mathcal {M}$	Linear regression	Direct instruction
High var / low bias $\mathcal {M}$	Neural networks	Discovery learning

Note that the last two rows are only examples of mechanisms that are *often* viewed as having high bias and low variance or high variance and low bias. See Doroudi (2020) for more details

### Substitution

The analogy operator as described above can be applied in cases that do not semantically appear to be analogies. For example, consider two papers that use different methods to achieve the same outcome; many of the entities and relations may be the same across the two representations, but the entity (or entities) representing the methods would be different. Colloquially we would probably not say there is an analogy between the two approaches. For this reason, we make a distinction between substitutions and analogies. A **substitution** operates exactly in the same way as an analogy, but it should be applied when it is more sensible. The analogous relation can be replaced with the substitutes relation for semantic clarity. Therefore, unlike the other operators, the distinction between the analogy and substitution operators is semantic. However, there are typically clear structural differences between the two. In a substitution, typically only one or a few entities and relations will change, and the rest will be identical across *P* and *L*. Moreover, a substitution is similar to what Gentner (1983) terms a literal similarity. Namely, Gentner (1983) suggests that the difference between a literal similarity and an analogy is typically that a literal similarity will involve a greater number of identical attributes (or unary relations).

Consider the following four scenarios that loosely describe different papers:

1. *Convolutional neural networks* are trained to classify histopathological images of breast tissue as benign or malignant (Spanhol et al., 2016).
2. *Support vector machines* are trained to classify histopathological images of breast tissue as benign or malignant (Aswathy & Jagannath, 2021).
3. *Human crowdworkers* are trained to classify histopathological images of breast tissue as benign or malignant (Eickhoff, 2014).
4. *Pigeons* are trained to classify histopathological images of breast tissue as benign or malignant (Levenson et al., 2015).

In cases 1 and 2, it would be a stretch to say that there is an analogy between “convolutional neural networks” and “support vector machines,” which are both machine learning algorithms that can be applied to the same classification tasks. Thus, here is a clear case of substitution. However, with case 4, even though one could argue a pigeon is being substituted for a machine learning algorithm, the idea of training pigeons and the idea of training machine learning algorithms both have long histories and are often used for different purposes. Thus, it seems more natural to say pigeons are analogical to neural networks or support vector machines in these scenarios (with the underlying abstraction being a learning agent). Pigeons and support vector machines have a lot fewer attributes in common than convolutional neural networks and support vector machines. Unlike pigeons, the latter two are both algorithms implemented in computer code that were designed specifically for classification tasks. Pigeons, on the other hand, are animals, fly, eat, and make sounds. Some attributes of pigeons are actually important for the training process but not shared by any standard machine learning algorithms, such as their hunger. While we might say getting hungry is analogous to the “reward seeking” or “loss minimizing” property of machine learning algorithms, there is no literal hunger in those algorithms.

Case 3 is less clear-cut. While human crowdworkers are also significantly different from machine learning algorithms, crowdsourcing is often used for tasks where state-of-the-art machine learning is not good enough or a machine learning engineer might want to compare the performance of their algorithm against crowdworkers. On the other hand, human crowdworkers and pigeons share a lot of similar attributes that are lacking in machine learning algorithms. These ambiguities point out that ultimately the decision of whether an analogy or a substitution applies is in the eyes of the beholder. In other words, the degree of overlap in attributes depends on what attributes are most salient to the researcher. If a crowdworker is seen as an alternative to artificial intelligence and its humanity is not at the forefront, then perhaps a substitution would apply. On the other hand, researchers interested in using pigeons’ visual properties as a substitute for human labelers (Levenson et al., 2015) could also see a substitution between crowdworkers and pigeons.

As mentioned earlier, Kang et al.’s (2022) analogical search engine looks for papers that overlap in terms of purpose with a researchers’ study (as represented in the form of a search query). However, if the purpose is virtually identical, then replacing one mechanism for another may often be a substitution, not an analogy, as seen in cases 1 and 2 above. In some cases, such as using pigeons vs. neural networks to classify images, swapping mechanisms may result in an analogy. On the other hand, when the purpose is only similar (but not identical), there is no guarantee that the purpose-mechanism relationship will be analogical across different papers.^3^ Thus, while Kang et al. (2022) find that their search engine is more likely to identify papers that trigger creative adaptations of the original idea (when compared to a standard keyword-based search engine), it is important to distinguish related work that might result in generating novel ideas and related work that actually has an analogical relationship with the present work.

Returning to Tu’s work on discovering a cure for malaria, she found that wormwood “showed some effects in inhibiting malaria parasites during initial screening, but the result was inconsistent and not reproducible.” Scouring over the relevant literature, she then identified a relevant sentence in Ge Hong’s fifth century *A Handbook of Prescriptions for Emergencies*: “A handful of Qinghao immersed in two liters of water, wring out the juice and drink it all” (Tu, 2015). Tu realized that while herbs are typically boiled, Ge’s recipe did not advocate for boiling it so perhaps the heat killed the active components in the wormwood. This led to a new method for extracting artemisinin from wormwood. To model this we would have to add entities to Fig. 5 that account for the method by which the drug is extracted. In that case, Ge’s method can be seen as a substitution for Tu’s original method. This substitution led to a drastic change in the research direction, eventually resulting in a cure for malaria.

**Fig. 7:**
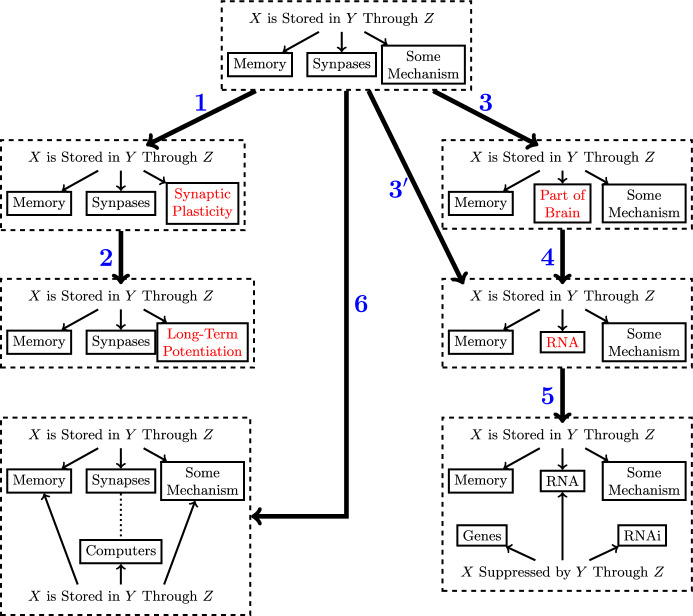
Example of literature search as a sequence of operators applied to a research question on how memory is stored in synapses

## Putting the pieces together

Now that we have seen the various operations that can relate two pieces of research to one another, it is worth discussing how these operations might be used in sequence over the scope of a research project. To do so, I provide a hypothetical example. As a disclaimer, the example is not from an area I have any expertise in; in fact, I encountered the relationships described below in the process of writing this paper (although not in the exact sequence described below). On the one hand, this suggests that the example may be oversimplified; on the other hand, perhaps it gives a somewhat authentic account of a non-expert navigating a new research field.

Suppose we are interested in conducting a literature review related to the question “how are memories stored in synapses?” This research question can be represented as “memories are stored in synapses through some mechanism” as shown at the top of Fig. 7. Some of the steps described below are also represented in Fig. 7; in those cases, I will mention the number of the step in parentheses. Operator names are italicized below. If the reader wants to assess their understanding of the operators (or perhaps assess the degree to which there could be subjectivity in which operators apply), the reader can guess which operator applies for each step of the figure before reading the rest of this section.

When embarking on this literature search process, we are likely already aware of some answers to the question. For example, “some mechanism” could be *reified* by “synaptic plasticity” (Step 1). But synaptic plasticity is quite broad and could be *reified* further by several more specific forms of plasticity, such as “long-term potentiation” (Step 2) and “long-term depression.” Further literature search might reveal a plethora of other mechanisms such as “protein synthesis,” “epigenetic mechanisms,” or “the standard model of synaptic consolidation.” However, these mechanisms are not necessarily mutually exclusive, perhaps leading to a revision of the question formulation to “memories are stored in synapses through a combination of X, Y, ...” (or some more hierarchical representation). On the other hand, some proposed mechanisms may be competing, like “the standard model of synaptic consolidation” and “multiple trace theory” (i.e., one can be *substituted* for the other). Moreover, we might realize that the “memory” entity can also be *reified* into particular kinds of memory, like “episodic memory” or “semantic memory.”

Searching the literature further may reveal that there are recent suggestions that memory is not (only) stored in synapses, but could be stored in sub-cellular materials. This might result in a *substitution* of certain molecules (e.g., “RNA”) for synapse (Step 3$'$). Alternatively, to keep our options open we may apply an *abstraction* of “synapse,” such as “parts of the brain” (Step 3). “Parts of the brain” can then be *reified* with many different entities, like “RNA” (Step 4). But it can also be substituted for regions of the brain where memories are stored, like the hippocampus. This may subsequently lead to the realization that rather than just asking how memories are stored, we should also be asking where memories are stored, leading to an *expansion* of the initial representation.

So far we have primarily considered literature that directly bears on the initial question. But sometimes surprising related works can also be discovered through *intersections*. For example, once we have established that RNA may be involved in memory, a colleague who is a molecular biologist might point out that there is an *intersection* with the literature on RNA interference (Step 5). Indeed, Smalheiser et al. (2001) noticed connections between a series of controversial 1960s studies on RNA-mediated memory transfer and RNAi; Smalheiser was a pioneer of literature-based discovery. We might then posit a relation that was neither present in our initial representation nor in related work: RNAi is potentially involved in the memory storage mechanism (i.e., “some mechanism” in our representation). Although it took over a decade, Smalheiser eventually found evidence to suggest that RNAi could indeed be involved in memory transfer (Smalheiser, 2017).

Finally, upon contemplating the initial representation further, the researcher may recognize an *analogy* to “how is memory stored in computer hardware?” (Step 6) or “how is memory stored in artificial neural networks?” Studying the literature in either of these areas may lead to the addition of new hypothesized mechanisms through an *interpretation* in light of the analogies. Notice that while in some cases a researcher notices an analogy when examining related literature, in other cases a researcher might think of an analogy, and then search for related literature. The related literature could either be about the analog (e.g., how memory is encoded in artificial neural networks) or about the analogy itself (Langille & Gallistel, 2020,e.g., how do theories of memory storage in the human brain relate to theories of memory storage in computer science). In the latter case, we have an *intersection* applied to the entire *analogy*.

## The typology in practice

In this section, we discuss some important considerations for how the representation and typology could be used in practice. In theory, an understanding of the various ways in which one piece of literature may relate to a research topic can inform directions in information retrieval and citation recommendation. Such systems could potentially represent papers in terms of entities and relations by using named entity recognition (Nadeau & Sekine, 2007) and relation extraction (Bach & Badaskar, 2007); they can also leverage a growing body of work on using knowledge graphs for information retrieval (Reinanda et al., 2020). The typology can then inform the kinds of relationships that such systems can explore and possibly recommend to users. However, we reiterate that there is no single way to represent a paper or single way of applying the operators to identify relationships to prior work. As noted above, the choice of what operators apply and hence which relationships to related works will be noticed depends on the view one takes of one’s work and related work. One way to potentially mitigate this challenge is by having users specify their current view of their work in terms of its representation, or perhaps by allowing them to simultaneously represent their work in multiple ways. Furthermore, recognizing that different researchers and papers will use slightly different terms to refer to identical or very similar entities and relations, search engines could try to treat semantically similar phrases as being identical or provide a pre-selected set of entities and relations that they recommend users use.

However, even if the representations of papers are completely aligned, the task of retrieving good analogies and abstractions may be computationally intractable in the worst case (Wareham et al., 2011). Indeed, in automated analogical search, simplifications are made to make finding potential analogies more tractable. For example, the MAC/FAC algorithm—which is rooted in structure-mapping theory—first finds several examples that have the most surface-level overlap in terms of relations and then identifies the analogy^4^ that is structurally strongest (Forbus et al., 1995). In Kang et al.’s (2022) analogical search engine, they look for papers that have a similar purpose, where similarity is measured by neural network embeddings rather than looking for a formally analogical structure. Although such algorithms may not be perfect, they could still potentially surface candidate analogies that would be given to a researcher who would ultimately identify when an analogy operator is applicable and useful.

Given the ongoing challenges in automated search, perhaps the typology would be more useful as a conceptual tool for researchers. Huang and Soergel (2013) found that “teaching users about the different kinds of topical relevance relationships may open their minds and make them better searchers and users of information.” Similarly, perhaps the typology presented here could be used as a tool to familiarize researchers with the different ways in which their research may relate to prior work, and how to use search tools to find such works. As mentioned before, simply representing one’s paper as a network of entities and relations may be a useful exercise to help researchers realize new insights about their research; future experimental studies could confirm whether this is true. Moreover, in discussing the potential value of their analogical search engine, Kang et al. (2022) mention the importance of “how deeply the human users can reflect on the retrieved analogs...and recognize how different notions of relevance may exist for their own problem context, despite potential dissimilarity on the surface” (p. 125). They suggest that “one approach to explaining relevance might be to surface a small number of core common features between an analog and a problem query” (p. 126). The representation presented here provides a natural way of showing users the potential relevance of related work. For example, when one searches for literature (even using a traditional search engine), representations could be generated on demand for the resulting papers such that they maximally align with the user’s query (at least in terms of number of entities and relations, if not in terms of higher-order relationships). Moreover, if the user specifies multiple research projects, a search engine could potentially represent each paper in terms of the representation that best aligns with each project.

## Conclusion

I have tried to make the case that literature search is a complex process that can influence and be influenced by research in a variety of ways. By describing research papers and projects in terms of concrete representations, we can formally articulate how different pieces of research might relate to one another. As discussed in the last section, this could have practical ramifications in terms of how search engines could better support the literature search process or how to design training for researchers to improve the way they approach literature search.

Beyond practical applications, the typology presented here could give us insight into the ways in which literature search might iteratively change the course of a research project as a sequence of operations. Although it goes beyond the scope of this paper, it might be worth briefly considering some of the ways in which a research project might be modified as a result of these operations. One form of modification is simply adding new entities and relations to *P* as a result of an expansion; we can view this as a natural extension of the **expansion** operator. Several other forms of modifications can fall under the category of logical inference (i.e., **deduction**, **induction**, and **abduction**). For example, in the black swans example, evidence of black swans triggers a modus ponens argument that proves the “all-swans-are-white” hypothesis is false, thereby changing *P*. Similarly, in Swanson’s ABC model, we can discern the presence of a new relation through the transitivity of the causal relation. If the representations are well-specified, one can imagine creating an inference engine that can automatically detect such changes in *P* after coming into contact with related work.

However, literature search cannot be considered in isolation from the other aspects of scientific discovery. Another form of modification to *P* might be the result of an **experimentation** operation, whereby a deduced relation is tested. We saw this both in the case of medical research that confirmed the causal link deduced by Swanson, and Tu’s experimental confirmation that wormwood can cure malaria. Finally, there is the **construction** operation, whereby a new entity or relation is created. Construction can result from either literature search (e.g., where an interpretation of some finding results in the discovery of a new finding, or where the expansion of an analogy results in an analogous entity that was not previously conceived of) or from research itself (e.g., the discovery of a new molecule or a new experimental finding). A thorough understanding of the processes of inference, experimentation, and construction is beyond the scope of this paper, but they begin to give us a hint as to how literature search is an iterative process that interacts with other aspects of the research process.

As pointed out by Swanson (1986), world 3 is also a world where scientific discovery takes place, by interacting with world 1 (the physical world) and world 2 (the subjective world of mental states). Philosophy of science should try to understand how these worlds interact in the process of scientific discovery; this paper is a step in that direction.

## Data Availability

N/A

## Footnotes

1 This can be formalized using the partial structures formalism mentioned above (Da Costa & French, 1990).

2 Gentner (1983) did not explicitly define an analogy in terms of an abstraction, but I believe it is useful to recognize that there is always implicitly an abstraction present, and in many cases, it might be useful to reason about what that abstraction is. Gentner (1983) further differentiates between abstractions, analogies, and literal similarities. These are differentiated by how many attributes and relations are shared between the two and the degree of abstractness of the entities (i.e., in an abstraction, entities are more abstract). While this is sensible, we allow for abstractions that are more concrete, so long as the entities in one representation are still *instances of* the entities in the other.

3 For example, one participant’s research question was how to “Grow plants better by optimizing entry of nanoparticle fertilizers into the plant” (p. 14). One paper identified by analogical search was about identifying plants by applying image analysis techniques to their leaves. It is not clear what the similar purpose is in this case, but regardless, the paper does not obviously share an analogical relationship with the research question. While this paper inspired a novel idea that the researcher thought would be relevant to her project, the relationship is captured by an intersection (through the “plant” entity) and possibly the application of interpretation and expansion operators.

4 Technically it looks for matches in terms of literal similarity to mimic people’s tendencies to find literally similar matches, but the algorithm could be easily modified to search for analogies.

## Declarations

### Code availability:

N/A
